# A rare cause of digital clubbing: pachydermoperiostosis

**DOI:** 10.11604/pamj.2016.25.194.10890

**Published:** 2016-11-25

**Authors:** Zeineb Alaya, Walid Osman

**Affiliations:** 1 Department of Rheumatology, Farhat Hached Hospital, Faculty of Medicine of Sousse, Sousse, Tunisia; 2 Department of Orthopaedics, Sahloul Hospital, Faculty of Medicine of Sousse, Sousse, Tunisia

**Keywords:** Pachydermoperiostosis, digital clubbing, thickening of the skin

## Image in medicine

A 35-year-old man of Tunisian origin complained of inflammatory arthralgia and he had noticed a progressive enlargement of his hands and feet as well as facial furrowing. On examination, he had thickening of the skin of the head and distal extremities and deep folds and furrows of the skin of the forehead (A), digital clubbing of fingers (B) and toes (C), spadelike enlargement of the hands and feet, hyperhidrosis of the hands and feet and seborrhea. Inflammation tests were disturbed. Radiographs showed periostosis of the long bones (D) and sacro-iliac osteosclerosis. After excluding secondary causes, primary hypertrophic osteoarthropathy also called pachydermoperiostosis (PDP) was established. This disease is characterised by thickening of the skin of the head and distal extremities, deep folds and furrows of the skin of the forehead, cheeks, and scalp, seborrhea; hyperhidrosis; periostosis of the long bones and digital clubbing. The treatment consisted in our case of analgesics and NSAIDs.

**Figure 1:**
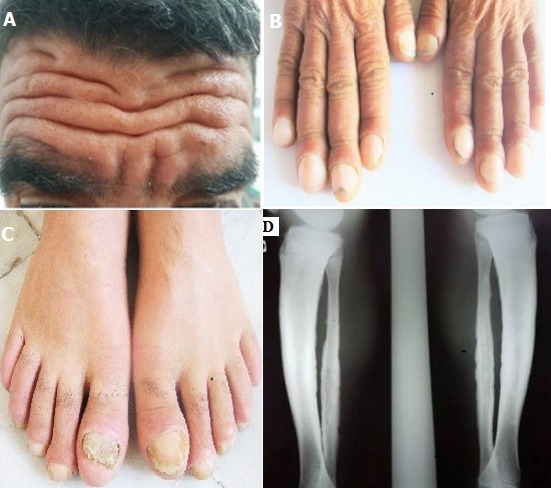
A) thickening of the skin of the head and deep folds and furrows of the skin of the forehead; B) digital clubbing of fingers; C) digital clubbing of toes; D) periostosis of the long bones

